# Lévy Walks Suboptimal under Predation Risk

**DOI:** 10.1371/journal.pcbi.1004601

**Published:** 2015-11-06

**Authors:** Masato S. Abe, Masakazu Shimada

**Affiliations:** Department of General Systems Studies, Graduate School of Arts and Sciences, The University of Tokyo, Tokyo, Japan; Univesidade de Lisboa, PORTUGAL

## Abstract

A key challenge in movement ecology is to understand how animals move in nature. Previous studies have predicted that animals should perform a special class of random walks, called Lévy walk, to obtain more targets. However, some empirical studies did not support this hypothesis, and the relationship between search strategy and ecological factors is still unclear. We focused on ecological factors, such as predation risk, and analyzed whether Lévy walk may not be favored. It was remarkable that the ecological factors often altered an optimal search strategy from Lévy walk to Brownian walk, depending on the speed of the predator’s movement, density of predators, etc. This occurred because higher target encounter rates simultaneously led searchers to higher predation risks. Our findings indicate that animals may not perform Lévy walks often, and we suggest that it is crucial to consider the ecological context for evaluating the search strategy performed by animals in the field.

## Author Summary

Moving agents should efficiently search for targets (e.g., food, prey, or specific locations) when lacking information about the location of the targets. For this random search problem, the Lévy walk hypothesis claims that Lévy walk movement patterns (i.e., each step length follows a distribution that is heavy-tailed) enable the searcher to capture more targets. However, most searchers may have antagonistic agents (e.g., predators) that can lead to death. Thus, the searcher needs to seek targets while avoiding encounters with antagonistic agents. Here, we show that the Lévy search strategy is less efficient in terms of total lifetime fitness when the predators are abundant, and especially when predators adopt a sit-and-wait strategy. Moreover, the results indicate that the life-cycle type of the searcher is an important fitness factor. These ecological aspects significantly influence the consequences of the random search. Therefore, it is critical to consider the ecological properties of searchers and other interacting agents when examining and estimating animal movements.

## Introduction

How should we move to search for targets when we have no information about their location? This is called the random search problem, which has attracted the attention of researchers in various fields [1]. The problem can be applied to various phenomena, including molecular-level movements within an organism, cell movements, movements of an individual animal, and the movement of robots [2–4]. For example, animals search their environment for food, prey, mates, and nesting locations, and DNA-binding proteins move around to find a specific DNA sequence to initiate gene expression. The search strategy is considered to evolve to be more efficient through the process of natural selection because successful searches increase fitness, especially at the individual level in animals.

The Lévy walk search (or foraging) hypothesis was proposed to solve the random search problem [5]. A Lévy walk is a special class of random walk models in which the probability function of step length *l* has a power-law tail: *P*(*l*)∼*l* ^−*μ*^(1<*μ*≤3), where *μ* is a power-law exponent, such that rare ballistic movements occur among a number of relatively short steps. Comparisons of the efficiency of random searches showed that a Lévy walk with *μ* ≈ 2 was a highly efficient search strategy in environments where patchy prey were sparsely distributed [1,5–7]. In dense environments, on the other hand, Lévy walks had almost the same efficiency as Brownian walks [6]. Therefore, the Lévy walk foraging hypothesis predicts that most animals should perform Lévy walks while searching unless there are abundant targets.

Although many empirical studies have reported that diverse organismal components and taxa (e.g., T cells, insects, and human beings) perform Lévy walks with *μ* ≈ 2 [1,3,8–15], several recent analyses demonstrated that some animals had various Lévy exponents *μ*, or they exhibited Brownian walks [13,14,16–18]. For example, rigorous statistical analyses of deer and bumblebees failed to provide strong evidence for Lévy walks [16]. Thus, the question changed from whether animals have Lévy walk movement patterns to when or why animals perform Lévy walks. In general, the diversity of organisms is the result of varying ecological and environmental factors as well as complex biotic interactions with conspecific and heterospecific individuals [19]. Theoretical reports of random searches have generally focused only on search efficiency to evaluate the fitness of the searcher [1,5–7,20–23]. Moreover, most of these studies paid little attention to other relevant ecological factors such as death rate with predation risk, interactions with other individuals, and the metabolic costs of foraging. A few studies have considered such factors [24–28], including predation risk [29–32]. In physics, Yuste et al. analyzed the survival rate of mortal random (Brownian) walkers surrounded by diffusing traps and revealed that the death in the course of motion dramatically affected the search efficiency [33]. Such a situation would be relevant to biological encounters. However, the relationships between search efficiency and predation risks in the context of Lévy walks are still poorly understood.

Here, we focus on the fact that search efficiency represents the probability of an encounter with anything existing in the environment. Highly efficient search strategies may correspond to more frequent encounters with predators, and thus higher death rates. Therefore, search efficiency, as defined in previous studies, may not reflect the actual fitness, because fitness is not only determined by the efficiency of searching for targets (i.e., benefits), but also by the death rate caused by predation (i.e., cost) [34]. Reynolds [30,31] reported that predation risk altered the optimal strategy, but did not consider predators [30] or the fitness of the searcher [31]. In this paper, we explicitly introduce predation risk and life-cycle types to the previous simulations, and extend the random search scenario to correctly estimate the fitness of a searcher to determine an animal’s optimal search strategy.

## Methods

### Basic assumption

First, we considered a searcher performing either the Lévy walk (hereafter, LW) or the Brownian walk (hereafter, BW) at movement velocity *v*_s_(= 1) in an environment in which patchy targets were sparsely distributed. Then, *N*_p_ predators were randomly placed in the environment as the initial condition. To explore the effect of the predators’ movements, we considered four cases with respect to the predators’ movement velocity *v*_p_/*v*_s_ = 0 (sit-and-wait); *v*_p_/*v*_s_ = 0.2 (slow); *v*_p_ / *v*_s_ = 1 (middle); and *v*_p_ / *v*_s_ = 5 (fast). If *v*_p_ > 0, we assumed that a predator performed LW with *μ* = 2 (or BW in S1 Text). For simplicity, we assumed that if the searcher encountered a predator, the searcher died from predation.

Second, if the death effect arising from encounters with predators was considered, search time became an important factor because the length of rest during searches was associated with fitness. Thus, each searcher had a maximum searching time, *T*_max_, that could be cut off by an encounter with a predator.

Finally, when considering the searcher’s fitness, we assume: (1) the fitness is the lifetime reproductive success (i.e., we analyze the number of offspring reproduced within the lifetime), (2) without alternation of generations (i.e., we do not take population dynamics into account), (3) a searcher has either one of two life-cycle types as described next. In the simplest case, finding a target directly led to increased fitness in a linear fashion (life-cycle type I). For example, when a female parasitoid wasp finds and attacks a host, and then searches for another target, we presume that its fitness increases linearly. Furthermore, when a male finds a female and mates, its fitness as a searcher also increases linearly. In contrast, animals characterized by life-cycle type II would need to survive until their reproductive stage *T*_max_ to obtain higher fitness. In life-cycle type II, individuals that die from predation prior to maturity have no offspring and have a fitness of zero.

### General relationship between fitness and the rate of encounter with targets and predators

Here, we show the general relationship between fitness and the rate of encounter with targets and predators as well as the robustness of *T*_max_ to our results. We denote the encounter rate with a predator per unit time Δ*T* as *γ*. The probability of an encounter with the predator at the *m*-th time unit is expressed as

$${\left(1-\gamma \right)}^{m-1}\gamma .$$ (1)

Therefore, when *T*_max_ is divided into *n* pieces by unit time Δ*T* (i.e., *T*_max_ = Δ*Tn*), the mean search time $\bar{T}$ is

$$\bar{T}=\sum_{m=1}^{n}\left\{m{\left(1-\gamma \right)}^{m-1}\gamma \right\}+n{\left(1-\gamma \right)}^{n}=\frac{1-{\left(1-\gamma \right)}^{n}}{\gamma },$$ (2)

where *n*(1−*γ*)^*n*^ indicates the case in which the searcher never encounters predators. When the mean number of encounters with predators for *n* is *k*, *nγ* = *k* and (1−*γ*)^*n*^ ≈ *e* ^−*k*^ for *γ* << 1 and a large *n*, thus

$$\bar{T}\approx \frac{n(1-e^{-k})}{k}.$$ (3)

#### Life-cycle type I

Here, we assume that an encounter with a target increases the fitness of the searcher by *α*. We denote the probability of the searcher encountering the target per unit time by *η*. The mean fitness of the searcher is

$$\phi =\alpha \eta \bar{T}\approx \frac{\alpha \eta n(1-e^{-k})}{k}$$ (4)

Hence, the relative fitness ratio of the Lévy walk to the Brownian walk is

$$\frac{\phi_{\text{LW}}}{\phi_{\text{BW}}}\approx \frac{\eta_{\text{LW}}k_{\text{BW}}(1-e^{-k_{\text{LW}}})}{\eta_{\text{BW}}k_{\text{LW}}(1-e^{-k_{\text{BW}}})}.$$ (5)

Thus, this value is independent of the maximum search time *T*_max_, and is determined by the balance between the mean number of encounters with predators at *T*_max_ and the search efficiency for targets per unit time.

#### Life-cycle type II

Next, we consider the case in which finding a target leads to a non-linear increase in fitness and stock targets until the reproductive stage (here *T*_max_). Therefore, the expected fitness is *nαη*(1−*γ*)^*n*^ ≈ *nαηe* ^−*k*^ for a large *n*. The relative fitness ratio of the Lévy walk to the Brownian walk is

$$\frac{\phi_{\text{LW}}}{\phi_{\text{BW}}}\approx \frac{\eta_{\text{LW}}e^{-k_{\text{LW}}}}{\eta_{\text{BW}}e^{-k_{\text{BW}}}}.$$ (6)

Thus, this value is also independent of the maximum search time *T*_max_. Unlike type I, in life-cycle type II, an encounter with targets does not directly impact fitness.

### Simulation

We calculated the fitness of LW and BW strategies in the ecological context using computer simulations because it is difficult to analytically derive the encounter rate in our relatively complicated setting, even though the analytical solutions were obtained in different scenarios under much simpler assumptions (e.g., Brownian walks, 1-D field, ideal gas model) [5,33,35,36,37]. Using the methods described previously [5,7,17], we simulated one searcher roaming in a 2-D environment in which some targets (e.g., food, hosts, mates) and predators were distributed. Although the species at higher trophic levels are lower in number in real ecosystems and the population we simulated seems unsustainable, we introduced only one searcher. This is because we focused on the fitness of a single searcher by picking it up from searcher’s population, and our main results must be robust if we introduce a number of searchers. The searcher had no prior information about the locations of both targets and predators, and wandered at a constant velocity *v*_s_ = 1 (per unit time) in a 2-D continuous field with length *L* ^2^ = 500×500 in which the boundary condition is periodic [7].

The LW was characterized by a distribution function *P*(*l*) ∼ *l* ^−*μ*^(1 < *μ* ≤ 3). In our simulations, we derived step lengths from the following equation to obtain LW, generating a uniform random number *u* (0 < *u* ≤ 1; except for *u* = 0):

$$l=l_{0}u^{{\left(1-\mu \right)}^{-1}},$$ (7)

where the minimum step length *l*_0_ is 1 [7]. For the BW simulation, to obtain an exponentially decaying distribution of the move length, each successive step length was drawn from a Gaussian distribution, where the mean was the minimum step length *l*_0_ = 1 and the variance was equal to 1 [7]. In LW or BW, after walking in a straight-line motion until reaching a step length *l*, the searcher turns in the angle drawn from a uniform distribution [−*π*,*π*].

The center of each patch was randomly scattered, and the radius of each patch was equal to 10. The number of targets and patches in the whole field was 1000 and 50, respectively. Each target was randomly assigned to a patch so that each patch had 20 targets on average. The targets were randomly distributed within the patch. In the initial state, *N*_p_ predators were randomly distributed in the whole area, i.e., the *x* and *y* position of each predator was independently drawn from a uniform distribution [0, *L*] (See S1 Text for the effects of initial conditions). *R*_t_ and *R*_s_ represented the radius of the targets and searcher, respectively, and $R_{\text{s}}^{\prime }$ and $R_{\text{p}}^{\prime }$ represented the radius of perception of the searcher and predators. If the distance between the searcher and a target was less than $R_{\text{t}}+R_{\text{s}}^{\prime }=1$, the searcher obtained the target, and the target disappeared. Then the step length of the searcher is truncated and recalculated, and the direction is drawn from a uniform distribution. After the searcher migrated a 500 path length, the depleted target regenerated to maintain the specified target density [17]. Similarly, the searcher died if the distance between the searcher and a predator was less than $R_{\text{s}}+R_{\text{p}}^{\prime }=1$. The mean free path *λ*, which represents the mean distance or travel time between patches or targets, is L22RN for the 2-D environment [7]. Hence, in our simulation, *λ*_patch_ = 2500 and *λ*_target_ = 125 for encounter distance *R* = 1. This is equivalent to the low-resource scenario of previous studies (e.g., [22]). The maximum search time *T*_max_ was 10^4^. To converge the results, the total time for a single parameter set (i.e., searcher’s movement pattern and density of predators) was 10^7^ for sit-and-wait, slow, or middle predator conditions, and 5×10^7^ for fast conditions. Then, *k*,*η*,*γ* were calculated, and the relative fitness was obtained using Eqs (5) and (6).

## Results

**Fig 1:**
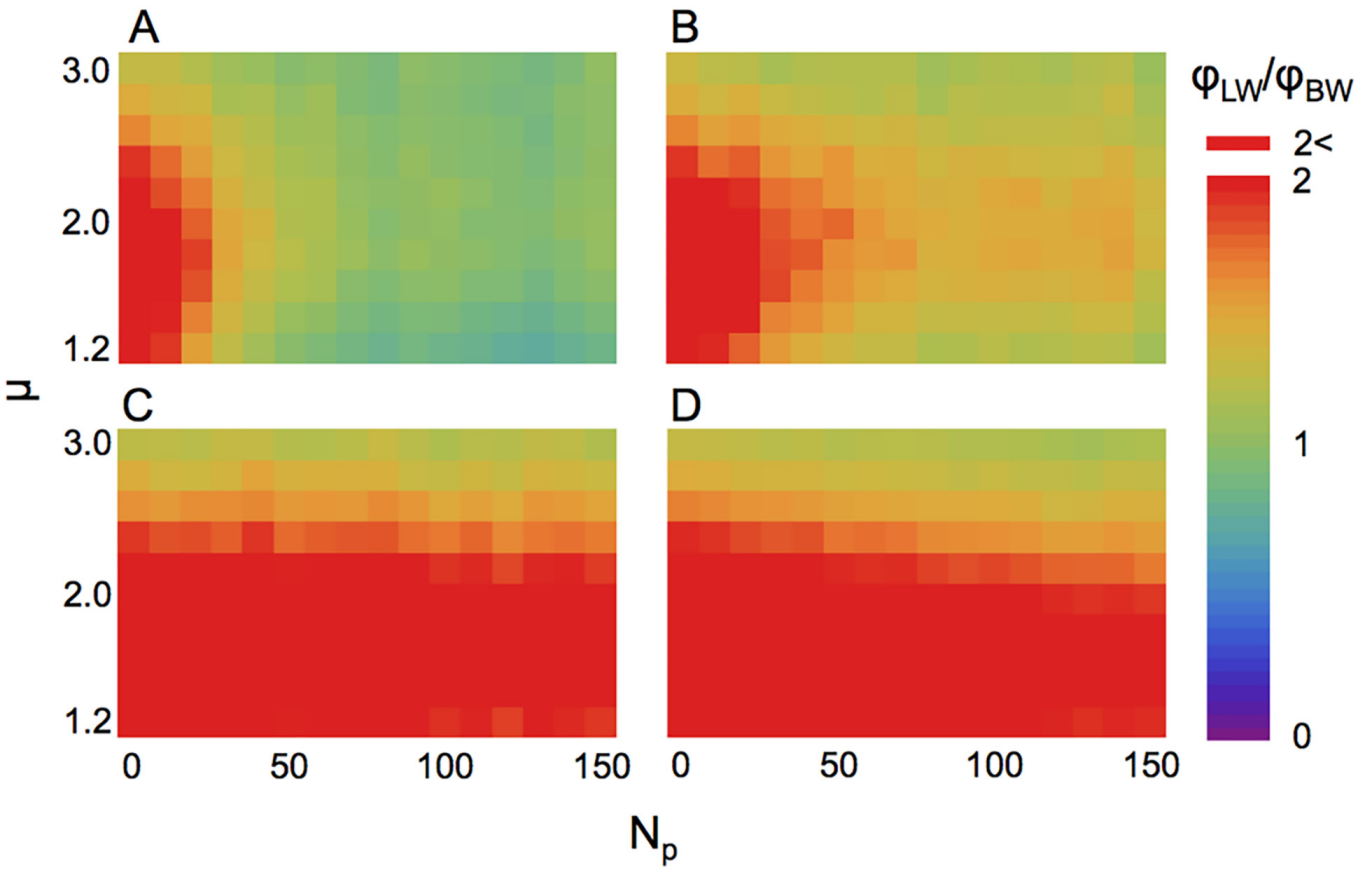
The relative fitness of a Lévy searcher with life-cycle type I. The strategy of predators is (A) sit-and-wait (*v*_p_ = 0); (B) slow Lévy walker (*v*_p_/*v*_s_ = 0.2); (C) middle Lévy walker (*v*_p_/*v*_s_ = 1); and (D) fast Lévy walker (*v*_p_/*v*_s_ = 5). The horizontal axis represents the number of predators introduced, and the vertical axis represents the Lévy index *μ* of the searcher. The total search time is 10^7^ for sit-and-wait, slow, and middle predator conditions and 5×10^7^ for fast predator conditions.

The results of the relative fitness (*ϕ*_LW_ / *ϕ*_BW_) calculation for life-cycle type I are presented in Fig 1. When predators were absent (*N*_p_ = 0), the relative fitness *ϕ*_LW_ / *ϕ*_BW_ was >2. Thus, LW with intermediate-level *μ* had the highest fitness, which was consistent with the findings of previous studies [5,7]. However, as the number of predators increased, the *ϕ*_LW_ / *ϕ*_BW_ ratio gradually declined to ~1 or slightly less than 1 when the predator strategy was sit-and-wait or slow LW (Fig 1A and 1B). In this case, a LW (*μ* ≈ 2) often performs best out of all LW’s. When the predator strategy was middle or fast LW, *ϕ*_LW_ / *ϕ*_BW_ was maintained at a high value, and LW could be an efficient strategy (Fig 1C and 1D).

**Fig 2:**
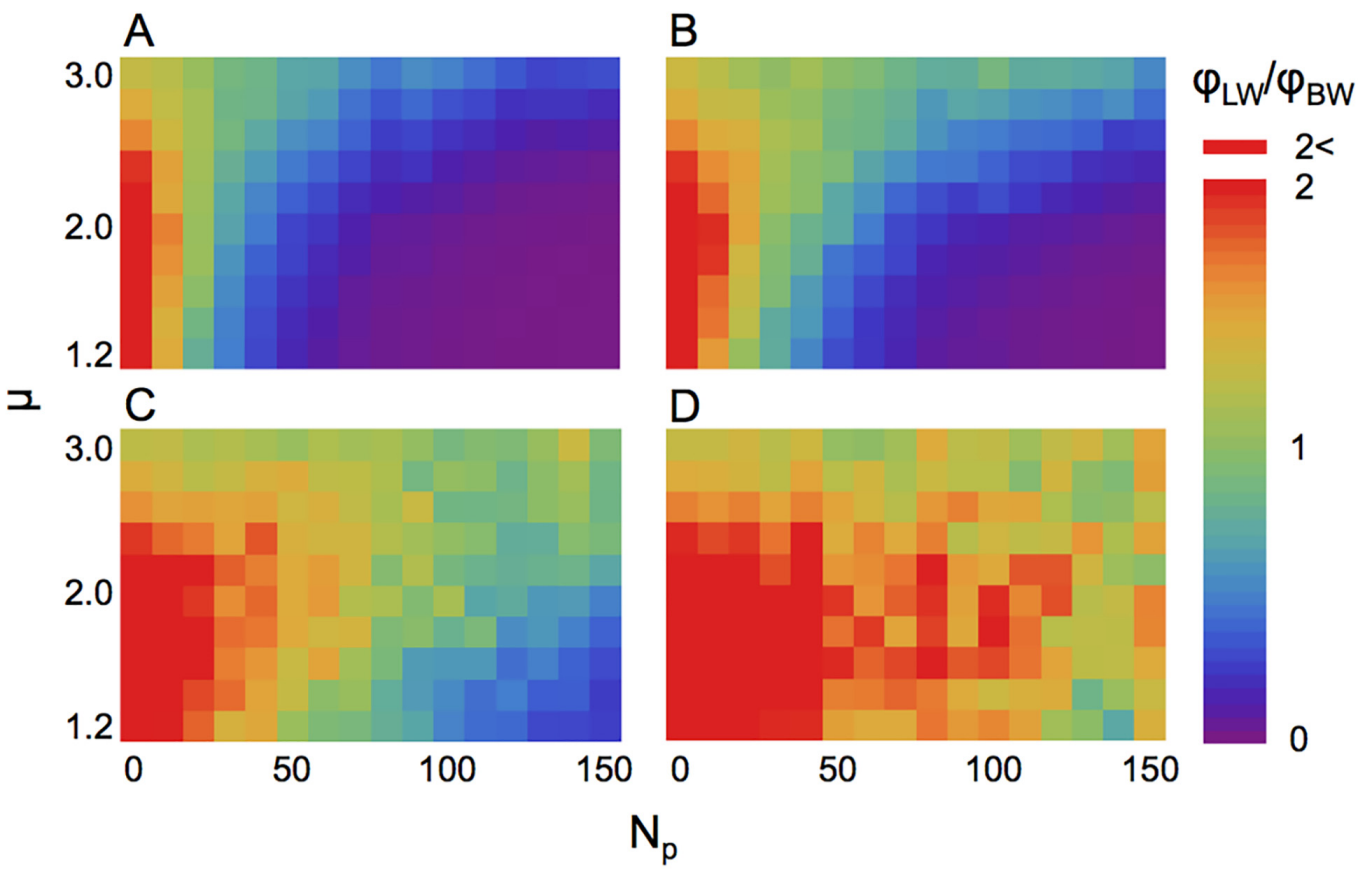
The relative fitness of a Lévy searcher with life-cycle type II. The strategy of predators is (A) sit-and-wait (*v*_p_ = 0); (B) slow Lévy walker (*v*_p_/*v*_s_ = 0.2); (C) middle Lévy walker (*v*_p_/*v*_s_ = 1); and (D) fast Lévy walker (*v*_p_/*v*_s_ = 5). The horizontal axis represents the number of predators introduced, and the vertical axis represents the Lévy index *μ* of the searcher. The total search time is 10^7^ for sit-and-wait, slow, and middle predator conditions and 5×10^7^ for fast predator conditions.

**Fig 3:**
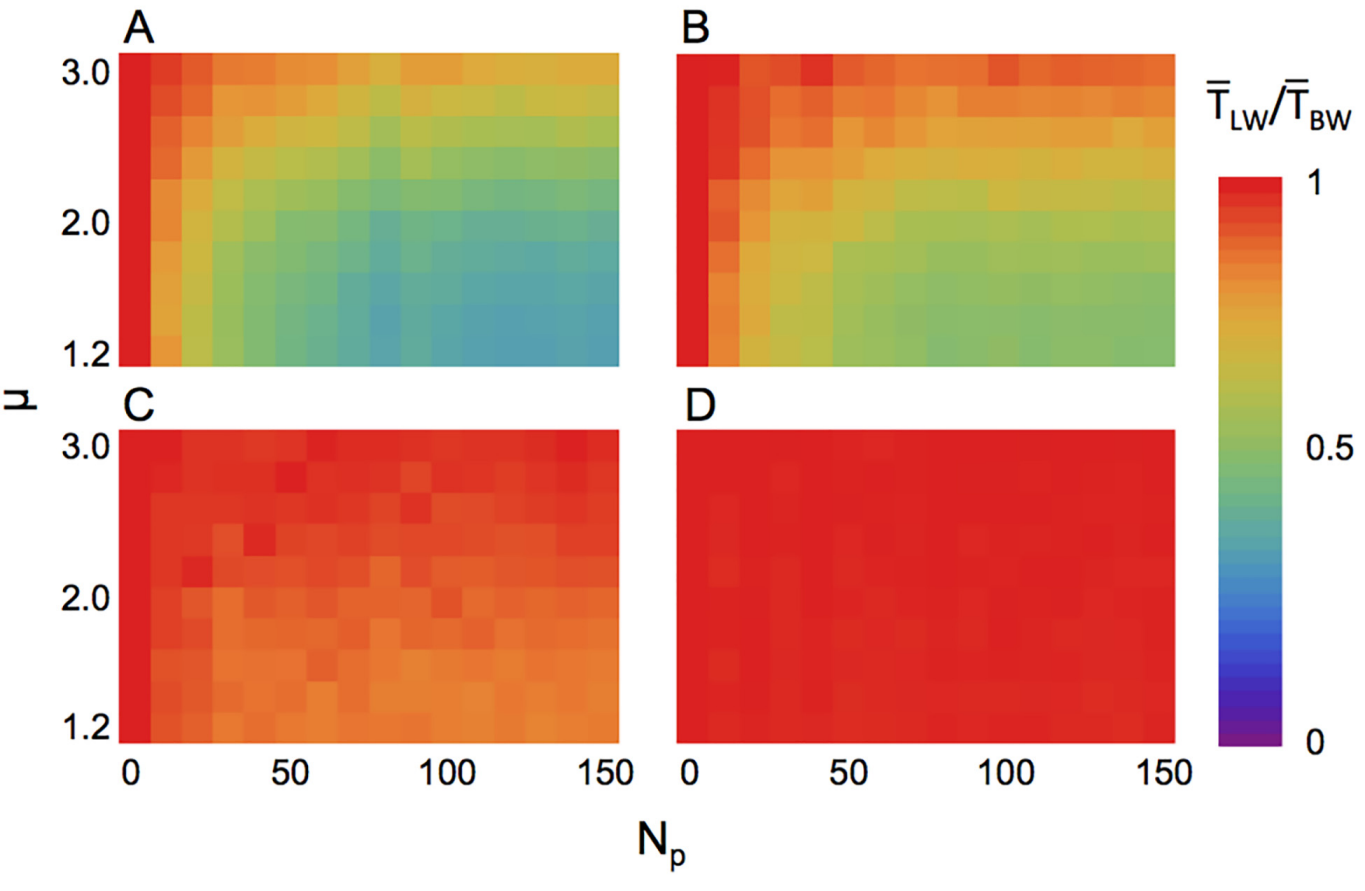
The relative mean search time changes depending on the density and velocity of predators. The strategy of predators is (A) sit-and-wait; (*v*_p_ = 0); (B) slow Lévy walker (*v*_p_/*v*_s_ = 0.2); (C) middle Lévy walker (*v*_p_/*v*_s_ = 1); and (D) fast Lévy walker (*v*_p_/*v*_s_ = 5). The horizontal axis represents the number of predators introduced, and the vertical axis represents the Lévy index *μ* of the searcher. The total search time is 10^7^ for sit-and-wait, slow, and middle predator conditions and 5×10^7^ for fast predator conditions.

Likewise, in the case of a searcher with life-cycle type II, the relative fitness *ϕ*_LW_/*ϕ*_BW_ decreased substantially as the number of predators increased when the predator strategy was sit-and-wait or slow LW (Fig 2A and 2B). Even when the strategy of predators was middle LW, *ϕ*_LW_/*ϕ*_BW_ decreased as the number of predators increased. These results were robust to other search strategies (i.e., correlated random walk or composite Brownian walk) (S1–S4 Figs) and to Brownian walk predators (S5 Fig). The relative fitness decreased because the searcher was likely to encounter a predator. The search time was shortened by death in a manner dependent on the search efficiency, and the relative mean searching time ${\bar{T}}_{\text{LW}}/{\bar{T}}_{\text{BW}}$depended on the search strategy (Fig 3). These results indicated that the LW strategy could lead to a high predator-encounter rate; therefore, BW could potentially be a risk-averting strategy.

**Fig 4:**
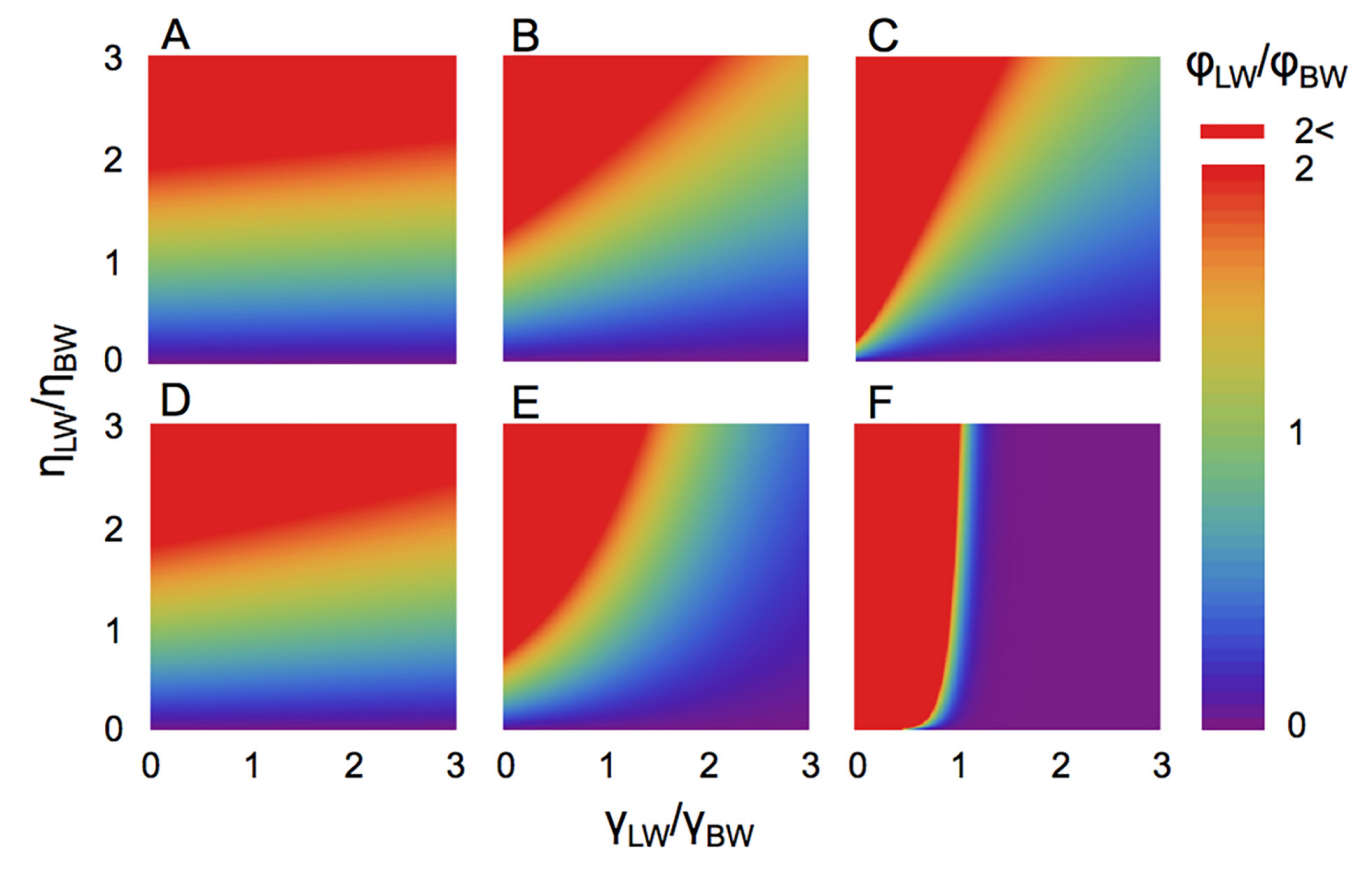
The relationship between relative encounter rates of targets and predators. Data in (A–C) and (D–F) correspond to life-cycle types I and II, respectively. (A and D) The lower encounter rate condition, *k*_BW_ = 0.1 (e.g., lower predator density or slower predators). (B and E) The middle encounter rate condition, *k*_BW_ = 1. (C and F) The higher encounter rate condition, *k*_BW_ = 10 (e.g., higher predator density or faster predators). The colors represent the fitness of LW (or any search strategies) compared to BW. The result shows that the life-cycle type and the three parameters, encounter rate to targets, that to predators and strength of predation pressure critically determine the fitness. When the strength of predation pressure is high (C and F), the LW obtains lower fitness even if the encounter rate with targets is high.

**Fig 5:**
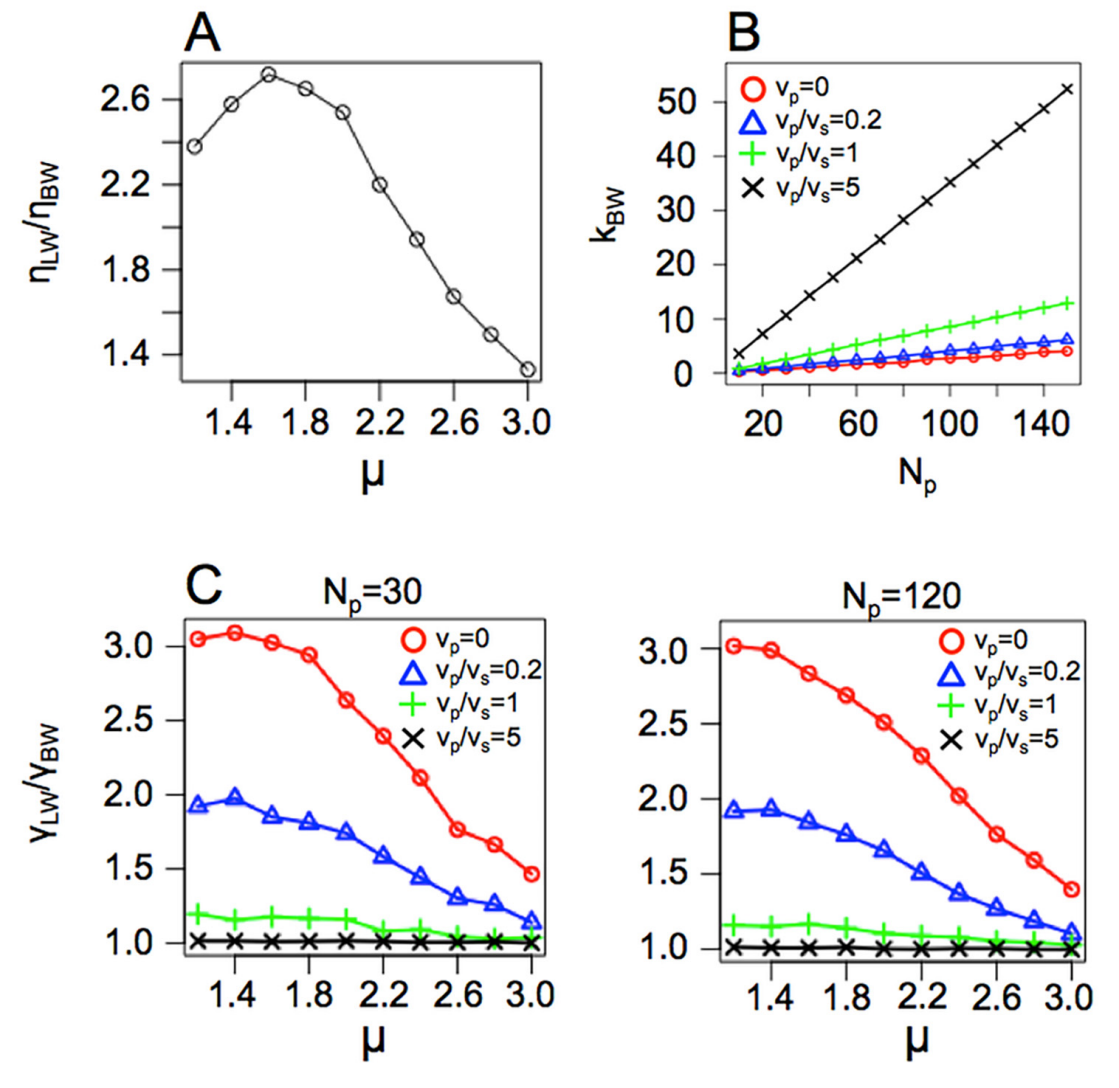
Encounter rates with targets and predators in our simulation setting. (A) The relative encounter rate with targets increases at an intermediate *μ*. (B) The mean encounter number of a BW searcher (*k*_BW_) for *T*_max_. As the number or velocity of predators increases, the encounter number increases. (C) The relative encounter rate with predators increases when the movement of a searcher approaches a straight line (i.e., smaller *μ*). However, the faster the movement of predators, the lower the rate of increase. Similar results for *N*_p_ = 30 (left) and *N*_p_ = 120 (right) indicate that the relative encounter rate does not change depending on predator densities.

To investigate these results, the relationship between the relative fitness and the encounter rate with targets and predators was examined (Fig 4). This result is not limited to our simulation results or to the relative fitness of LW or BW, but it describes a general trend. The relative encounter rates with targets and predators and the expected encounter number of BW for our simulation are presented in Fig 5. When the encounter rate with predators was low (i.e., low *k*_BW_), the fitness of random search strategies clearly depended on the encounter rate with targets (Fig 4A and 4D). Hence, LW had higher fitness in our simulation (Fig 5A). On the other hand, for intermediate or high *k*_BW_, fitness also changed depending on the encounter rate with predators (Fig 4B, 4C, 4E and 4F). Furthermore, fast predators displayed the same high predator encounter rates of high *k*_BW_ and γLWγBW≈1 (Fig 5B and 5C). Thus, LW had higher fitness under the fast-predator conditions for life-cycle type I and almost equal fitness for life-cycle type II. Similarly, the fitness of other random search strategies was determined by the encounter rate with targets and predators. The degree of encounter rate improvement not only depends on the search strategy, but also on the distribution or density of the targets [5,7], suggesting that the conditions for the optimal search strategy are complex.

## Discussion

### Predators alter the optimal searching strategy

Our results revealed that the random search strategy affected the death rate arising from predation, and that trade-offs could occur between foraging efficiency and predation risk. In nature, animal species have different ecological traits or interactions associated with their foraging behavior [34,38,39]. Considering such ecological factors, optimal foraging theory, as it currently exists, successfully predicts various types of animal behaviors from the viewpoint of maximizing fitness through natural selection [38,39]. However, previous studies of random search movements have only focused on foraging (i.e., search efficiency for targets), which may be unrealistic when considering the diversity of ecological characteristics and biotic interactions in nature. Lima et al. [34] reported that animals performed more efficient strategies in response to ecological factors, including risks, with such trade-offs. Our simulations predicted that where predators were abundant, a searcher performing a LW might have lower fitness depending on its ecological characteristics and those of the predators. This suggests that the optimal search strategy may change. Therefore, the parameter range in which the LW is advantageous may be narrower than previously estimated (Fig 4). The mechanism explaining these dynamics was that LWs not only increased the encounter rate with targets, but also with predators, which shortened the lifespan in exchange for the capture of more targets. The rare ballistic movements of LWs led to the high encounter rate with predators (Fig 5C), and this effect has been reported as a high encounter rate of a straight line motion with randomly distributed destructive targets [7] or new targets [35]. In the presence of predators, a searcher was confronted with conditions similar to the destructive search problem, because encounters with predators resulted in the death of the searcher. Although we assume the ecological context in this paper, such searching-avoiding trade-offs in the random search problem that we revealed here may occur in other contexts such as protein-DNA interactions [2,40].

Previous studies analyzing the predation effect on search strategies focused on the predation risk within a patch [30, 31], and reported that the predation risk could alter the optimal time spent for intensive searches if the predation risk increased as the time spent within a patch increased. In contrast, we concentrated on the predation risk in a whole area and predicted the fitness ratio between LW and BW by calculating the encounter rate with targets and predators. Also, Reynolds simulated the moving preys searched by one predator [31], and the study discussed that the prey movement patterns were determined by their foraging and not by cost of predation when predators are fast. This idea is consistent with our results for life-cycle type I (especially in Fig 1D), but we defined the fitness based on life-cycle and simulated the tri-trophic system consisting of targets, searchers, and predators. Consequently, we revealed the general effect of predation risk on search strategy (Fig 4).

To disentangle the effects of density, radius, and velocity of a searcher or predators on the relative fitness, we refer to analytical results of simple situations. Hutchinson et al. [35] and Dusenbery [37] reviewed the analytical results for the encounter rate of two kinds of straight motion agents (e.g., target and searcher, or searcher and predator) in 2-D and 3-D. In this case, the encounter rate is proportional to both the density of agents and encounter distance (i.e., $R_{\text{s}}+R_{\text{p}}^{\prime }$ in our model). In our results, the density of predators is an important factor that can determine the relative fitness. In Fig 5C, the left (low density) and right (high density) figures are almost identical because the effect of predator’s density in the ratio of encounter rate γLWγBW is cancelled out. Although the movements in our simulation are not straight motions but LW or BW, the proportionality of density effects on encounter rate could be common. Therefore, the density of predators can affect only the number of encounters to predators *k*. The ratio of encounter rate depends on the characteristic of movements (i.e., LW or BW, and velocity) rather than the density of predators. Additionally, the radius of searcher and predators, that is, encounter distance can be also the same effect as the density of predators in our results because the encounter rate can be proportional to the encounter distance.

The encounter rate in 2-D of a stationary searcher and straight motion predators with constant speed *v*_p_ is 2*ρR*_s_ *v*_p_ where *ρ* is the density of predators [37], and that of a straight motion searcher and predators with the speed *v*_s_ = *v*_p_ is 8*ρR*_s_ *v*_p_ / *π* [35]. Hence, the ratio of the encounter rates is 4 / *π*. This is consistent to our result for the ratio of encounter rate of BW (i.e., like a stationary searcher) and LW with small *μ* (i.e., like a straight motion searcher) under the presence of LW predators (i.e., like straight motion predators) in the case of *v*_p_/*v*_s_ = 1 (green line in Fig 5C). In the case of *v*_p_/*v*_s_ = 0, 0.2, 5, the movement of the faster individuals has a large effect on the encounter rate [37]. Therefore, compared with BW, LW in *v*_p_/*v*_s_ = 0, 0.2 has the high encounter rate with predators (Fig 5C). Additionally, the analytical result for 3-D conditions is similar to that for 2-D [37]. Thus, our conclusion could be applied to 3-D such as prey-predator interactions of planktons in lakes or ocean.

Moreover, a recent study proposed a framework for encounter rates that are derived from an arbitrary trajectory of a searcher and immobile targets using an encounter kernel [41]. The combination of this technique and our results for general relationship between encounter rate and fitness (Fig 4) may provide the general framework integrating movements and fitness. This could give us the information about fitness directly from the trajectory and distribution of targets.

### Relation to empirical studies

Many empirical studies have reported that the movement patterns of animals, from insects to human beings, are expressed as LWs with *μ* ≈ 2 [1]. However, the power-law exponents fitted to movement patterns sometimes ranged from 2 to 3 [1], suggesting that movement patterns may be diverse. Additionally, the data best fitted to the exponential decay distribution (i.e., BWs) has also been reported [13,14,16,18,42,43]. In theoretical studies, the first attempt reported that LWs with *μ* ≈ 2 were optimal for targets that can be revisited (i.e., non-destructive) or those that are extremely patchy [5]. Moreover, LWs with *μ*→1 (i.e., straight movement) were the optimum for randomly distributed destructive targets. After the study, the results of several versions of simulations suggested that LWs with 1 < *μ* ≤ 2 are more efficient depending on the prey distribution and other factors [20,21,26]. For the power-law exponent *μ* > 3 (i.e., BW), it has been theoretically reported that the foraging efficiency is similar to LW under high-resource conditions [6]. Our results suggests that under high predation risk, animals with power-law exponents close to three have higher fitness than *μ* ≈ 2 or *μ* < 2 (Figs 1 and 2), and those under intermediate predation risk, LW with 2 < *μ* < 3 also benefit. Therefore, it can be an alternative explanation for the diversity of power-law exponents.

### Mechanisms for Brownian movement

There is a question of whether movements in animals are spontaneous patterns for adaptation or a reflection of interactions with targets or complex environments [42]. de Jager et al. experimentally explained Brownian movement patterns of mussels by truncations resulted from encounters with conspecific individuals, which is the original mechanism of Einstein’s collision-induced BWs [42,44]. In contrast, our findings suggested that spontaneous BWs were beneficial, and this conclusion is supported by the fact that the pattern can spontaneously change depending on internal physiological states [45,46]. Of course, our hypothesis does not contradict the claim of de Jager et al., because the spontaneous LW pattern has higher efficiency in the absence of risk.

### Changing search strategy

Furthermore, our results suggest that animals can change their search strategy according to their developmental stage or in response to predator cues. For example, a juvenile individual under high predation pressure might adopt the BW strategy to avoid predator encounters, but an adult might adopt the LW strategy to obtain more targets in the absence of predators or under low predation pressure. In smaller scale responses, when an individual receives a chemical cue (kairomone) that indicates the presence of a predator, switching the internal pattern from LW to BW may represent an adaptive searching strategy, because the stochastic or random pattern can arise from internal processes [32,46–49]. Although such switching strategies depending on the target distribution have been investigated [9,13,14,42,50], the response to predators is less understood [51] and may be a topic for further study.

### Limitation of encounter event with predators

We introduced fitness determined not only by search efficiency but also by predation risk into the random search scenario unlike previous studies. In our assumption, the encounter with predators leads to death of the searcher with probability 1. This means that the first encounter with the predator is crucial for the searcher, and seems to be more dangerous for the searcher than the actual situation in nature because the encounter in nature does not always lead to death. If the probability is less than 1 and the searcher survives the encounter with a predator, then the searcher starts to move from the position near the predator as the simplest assumption. In this case, the problem reduces to the difference of initial positions. The result of effects of initial distance between the searcher and the nearest predator suggests that the short distance decreases the relative encounter rate with predators γLWγBW when the predator’s strategy is sit-and-wait (S6 Fig). Therefore, the Lévy walk strategy can temporarily benefit from departing from the close predator [30], indicating that the switch between strategies could be more efficient.

However, considering the biological plausibility, animals would not start to move around in a random manner immediately after an encounter with a predator. Instead, the searcher must depart from sit-and-wait predators using the information about the location of the predator in a deterministic manner, or dash to a safe area (e.g., bushes) to hide from moving predators and wait for the predator to leave. The predators would leave the location after some giving-up-time. The encounter event with a predator seems to transcend the simple framework of the random search problem. However, if the searcher starts random searches after fully departing from predators, the condition should not change much. Thus, probability 1 can represent several situations of prey-predator interactions.

Although we can use the probability of the survival for simplification, more complex interactions between prey and predator should occur in nature. Some empirical studies have reported the variability in predator avoidance [52,53], and theoretical studies have solved the pursue-evasion problem [36,54]. Although the issue of how the random search problem relates to such complex interactions is an interesting one, the relationship is poorly understood at present, and awaits further study.

### Estimation of animal movements

Tracking animal movements over a prolonged period of time (biologging) is a method developed within the last decade that can lead to the understanding of dynamic phenomena ranging from the individual level to population and community levels [55,56]. Because the differences in searching strategies influence diffusiveness and movement patterns of animals, it is crucial to identify the search strategy that animals adopt in a natural environment. The tracking of animal movements within the framework of movement ecology requires information on biotic interactions and interactions between individual animals [57–59]; therefore, the context in our model should be common to various animal species in nature, because most animals are exposed to predation pressures or to the risk of death during searching. Likewise, predators may be exposed to the risks of higher-order predators. For further investigation, it will be interesting to explore the complex dynamics via the interactions between movement and population dynamics. Thus, considering ecological factors can lead to a fruitful understanding of the dynamics at various scales.

## Supporting Information

S1 Text Analysis of other strategies and initial conditions. (DOCX)

S1 Fig The relative fitness of a CRW searcher with life-cycle type I. The strategy of predators is (A) sit-and-wait (*v*_p_ = 0); (B) slow Lévy walker (*v*_p_/*v*_s_ = 0.2); (C) middle Lévy walker (*v*_p_ / *v*_s_ = 1); and (D) fast Lévy walker (*v*_p_/*v*_s_ = 5). The horizontal axis represents the number of predators introduced, and the vertical axis represents the shape parameter *ρ* of the searcher. The total search time is 10^7^ for sit-and-wait, slow, and middle predator conditions and 5×10^7^ for fast predator conditions.
(TIFF)

S2 Fig The relative fitness of a CRW searcher with life-cycle type II. The strategy of predators is (A) sit-and-wait (*v*_p_ = 0); (B) slow Lévy walker (*v*_p_ / *v*_s_ = 0.2); (C) middle Lévy walker (*v*_p_ / *v*_s_ = 1); and (D) fast Lévy walker (*v*_p_ / *v*_s_ = 5). The horizontal axis represents the number of predators introduced, and the vertical axis represents the shape parameter *ρ* of the searcher. The total search time is 10^7^ for sit-and-wait, slow, and middle predator conditions and 5×10^7^ for fast predator conditions.
(TIFF)

S3 Fig The relative fitness of a CBW searcher with life-cycle type I. The strategy of predators is (A) sit-and-wait (*v*_p_ = 0); (B) slow Lévy walker (*v*_p_/*v*_s_ = 0.2); (C) middle Lévy walker (*v*_p_ / *v*_s_ = 1); and (D) fast Lévy walker (*v*_p_ / *v*_s_ = 5). The horizontal axis represents the number of predators introduced, and the vertical axis represents the giving-up length of the searcher. The total search time is 10^7^ for sit-and-wait, slow, and middle predator conditions and 5×10^7^ for fast predator conditions.
(TIFF)

S4 Fig The relative fitness of a CBW searcher with life-cycle type II. The strategy of predators is (A) sit-and-wait (*v*_p_ = 0); (B) slow Lévy walker (*v*_p_ / *v*_s_ = 0.2); (C) middle Lévy walker (*v*_p_/*v*_s_ = 1); and (D) fast Lévy walker (*v*_p_ / *v*_s_ = 5). The horizontal axis represents the number of predators introduced, and the vertical axis represents the giving-up length of the searcher. The total search time is 10^7^ for sit-and-wait, slow, and middle predator conditions and 5×10^7^ for fast predator conditions.
(TIFF)

S5 Fig The relative fitness of a LW searcher in the presence of BW predators. The strategy of predators is (A, D) slow Brownian walker (*v*_p_ / *v*_s_ = 0.2); (B, E) middle Brownian walker (*v*_p_/*v*_s_ = 1); and (C, F) fast Brownian walker (*v*_p_ / *v*_s_ = 5). The horizontal axis represents the number of predators introduced, and the vertical axis represents the giving-up length of the searcher. The total searching time is 10^7^ for sit-and-wait, slow, and middle predator conditions and 5×10^7^ for fast predator conditions.
(TIFF)

S6 FigSimulation results for the relationship between the initial distance *d*_n_ and encounter rate with predators.The horizontal and vertical axis represents the Lévy index *μ* of the searcher and the relative encounter rate γLWγBW with predators, respectively. When predators adopt a sit-and-wait strategy, the close distance to the nearest predator can lead to make γLWγBW low. The number of predators is 100 and other parameters are the same as those of the main results.(TIFF)
