# Supplementary material for: Lévy Walks Suboptimal under Predation Risk
Source: PLoS Comput Biol. 2015 Nov 6;11(11):e1004601. doi: 10.1371/journal.pcbi.1004601 (PMC4636162; doi:10.1371/journal.pcbi.1004601)
Supplement: S1 Text — (DOCX) [file pcbi.1004601.s001.docx]

**Supporting Information (S1 Text)**

*Lévy Walks Suboptimal under Predation Risk*

**1. Analysis of other search strategies under predation risk**

We analyzed the fitness of a searcher performing the alternative search strategy correlated random walk (CRW) and composite Brownian walk (CBW) proposed by previous studies [1, 2]. CRW is positively correlated with previous direction when the direction changes, and we selected the directional change from a wrapped Cauchy distribution with shape parameter [1, 3, 4]. For , CRW corresponds to BW, and CRW becomes a straight-line movement as approaches 1. The directional change is drawn from the following equation

(1)

where $u$ is a uniform random variable from 0 to 1. Each step length between two successive directional changes is drawn from a normal distribution where the mean value is the minimum step length 1 and the variance is 1.

The CBW is composed of BW and straight motion. CBW performs a straight motion until it encounters a target, at which point it changes to BW. Then, CBW returns to straight motion after moving a certain distance (i.e., giving-up length). Therefore, CBW exploits targets within a patch and then explores a new patch. Benhamou [2] reported that the step length distribution of CBW was similar to that of LW as a result of interaction with the environment (i.e., targets) and that CBW had high search efficiency.

The other parameters and assumptions for calculating relative fitness of CRW and CBW, namely and , are the same as those of the LW simulation in the Main Text. The results are presented in S1–S4 Figs. As was the case for LW, BW outperformed the CRW and CBW when predators were abundant.

**2. LW searcher vs. BW predators**

If BW has higher fitness than LW under predation risk, a predator for a searcher should also perform the BW. Here, we analyzed the fitness of LW and BW under BW predators (S5 Fig). The simulation method is the same as that under LW predators. A LW searcher has lower fitness under some BW predators because fast or middle BW with lower dispersal ability plays a role similar to that of a slow LW predator. This indicates that if the numbers of a top predator in a food web increase, the presence of that top predator may affect the strategy of animals in the second and third levels from the top by a cascade effect.

**3. Effect of initial condition on the fitness**

The initial positions of predators in the main results were randomly determined, i.e., following a uniform distribution. In this section, we examine the effect of initial conditions on fitness. We assumed that the distribution of food and predators is the same with the condition mentioned in the Methods section of the main text, but the searcher starts to move from a position near a predator. Although this condition seems extraordinary, the result would be insightful for understanding the effect of death rate < 1 on fitness. We set the fixed initial distance *d*_n_ between the searcher and nearest predator 2.5 (close) or 25 (normal) while keeping the distribution of predators random. The *d*_n_ = 25 of the normal condition indicates the average distance between the searcher and nearest predator at an initial state when the 100 predators are distributed randomly (See [5] for the derivation), suggesting the normal condition reflects the condition of our main results. S6 Fig shows the close initial distance reduces the relative encounter rate with predators when the predator’s strategy is sit-and­-wait. This stems from the fact that when adopting the BW strategy an encounter with the nearest neighbor can occur readily due to the intensive search of the surrounding area of the searcher. On the other hand, in the LW strategy the searcher tends to move away from the nearest predator. The results suggest the efficiency of the Lévy walk under the condition where the searcher is extremely close to the predator. Conversely, when the predator’s movement is fast, does not change much because the predator tends to move away from the searcher.

**References**

1. Bartumeus F, da Luz MGE, Viswanathan GM, Catalan J (2005) Animal search strategies: a quantitative random-walk analysis. Ecology 86: 3078–3087.
2. Benhamou S (2007) How many animals really do the Lévy walk? Ecology 88: 1962–1969.
3. Haefner JW, Crist TO (1994) Spatial model of movement and foraging in harvester ants (*Pogonomyrmex*)(I): The roles of memory and communication. J Theor Biol 166: 299–313.
4. Zollner PA, Lima SL (1999) Search strategies for landscape-level interpatch movements. Ecology 80: 1019–1030.
5. Clark PJ, Evans FC (1954) Distance to nearest neighbour as a measure of spatial relationships in populations. Ecology 35: 445–453.
