# Supplementary figures and images for: Lévy Walks Suboptimal under Predation Risk
Source: PLoS Comput Biol. 2015 Nov 6;11(11):e1004601. doi: 10.1371/journal.pcbi.1004601 (PMC4636162; doi:10.1371/journal.pcbi.1004601)

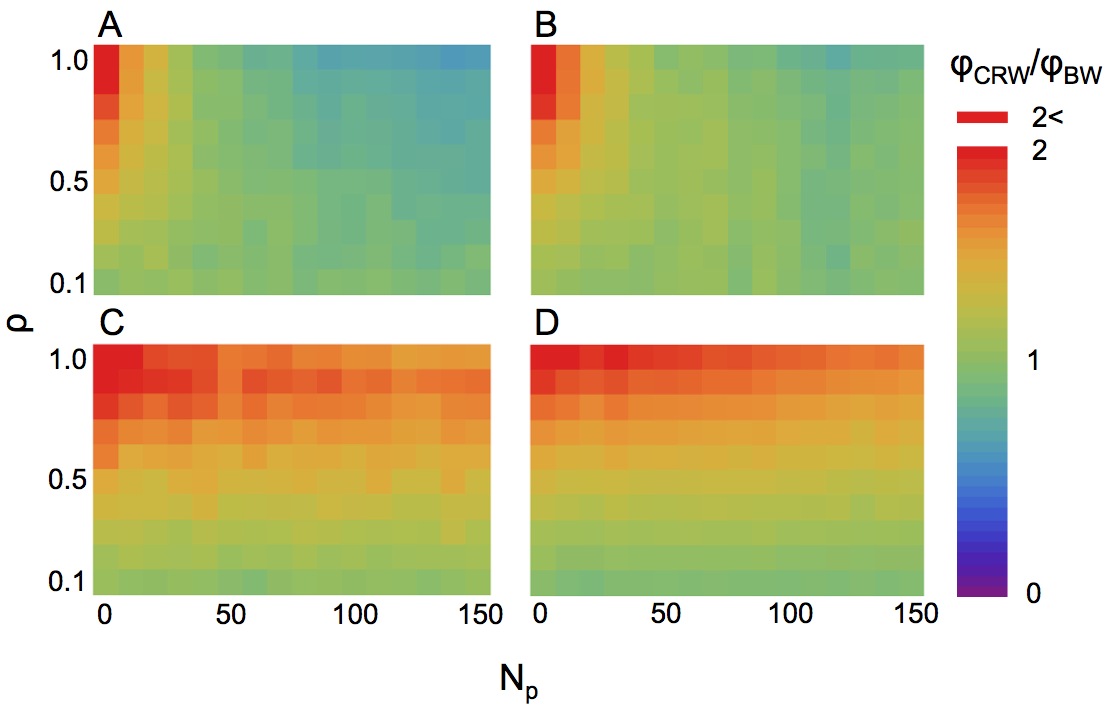

Supplement: S1 Fig — The strategy of predators is (A) sit-and-wait (v p = 0); (B) slow Lévy walker (v p/v s = 0.2); (C) middle Lévy walker (v p / v s = 1); and (D) fast Lévy walker (v p/v s = 5). The horizontal axis represents the number of predators introduced, and the vertical axis represents the shape parameter ρ of the searcher. The total search time is 107 for sit-and-wait, slow, and middle predator conditions and 5×10^7^ for fast predator conditions. (TIFF) [file pcbi.1004601.s002.tiff]

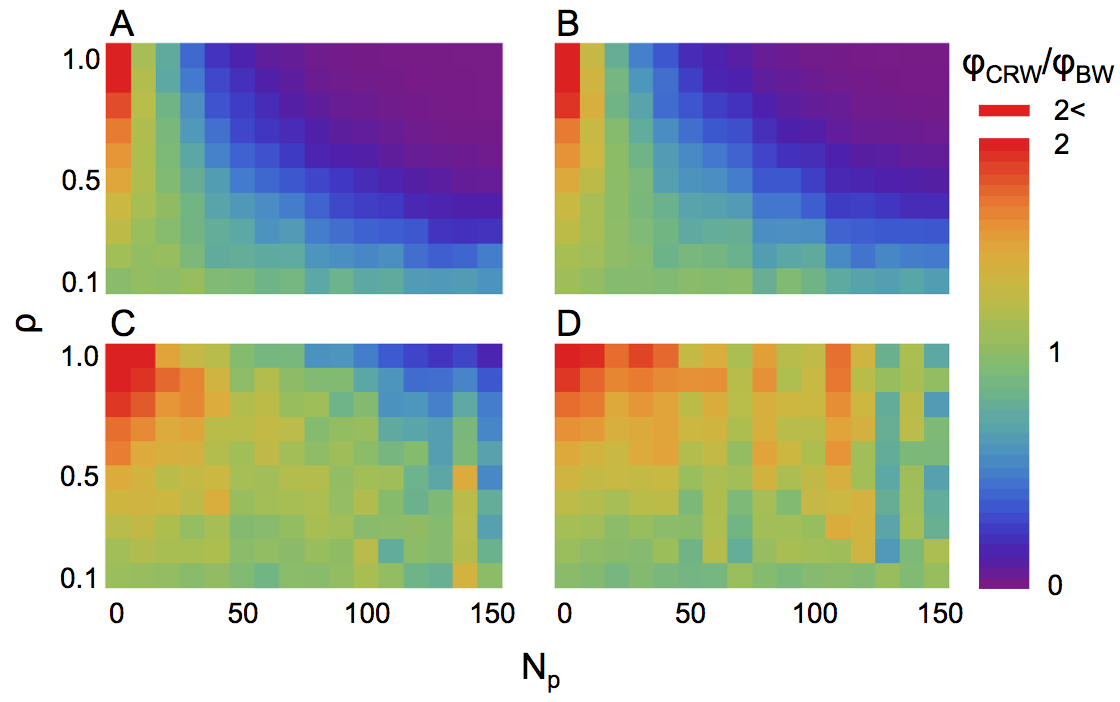

Supplement: S2 Fig — The strategy of predators is (A) sit-and-wait (v p = 0); (B) slow Lévy walker (v p / v s = 0.2); (C) middle Lévy walker (v p / v s = 1); and (D) fast Lévy walker (v p / v s = 5). The horizontal axis represents the number of predators introduced, and the vertical axis represents the shape parameter ρ of the searcher. The total search time is 107 for sit-and-wait, slow, and middle predator conditions and 5×10^7^ for fast predator conditions. (TIFF) [file pcbi.1004601.s003.tiff]

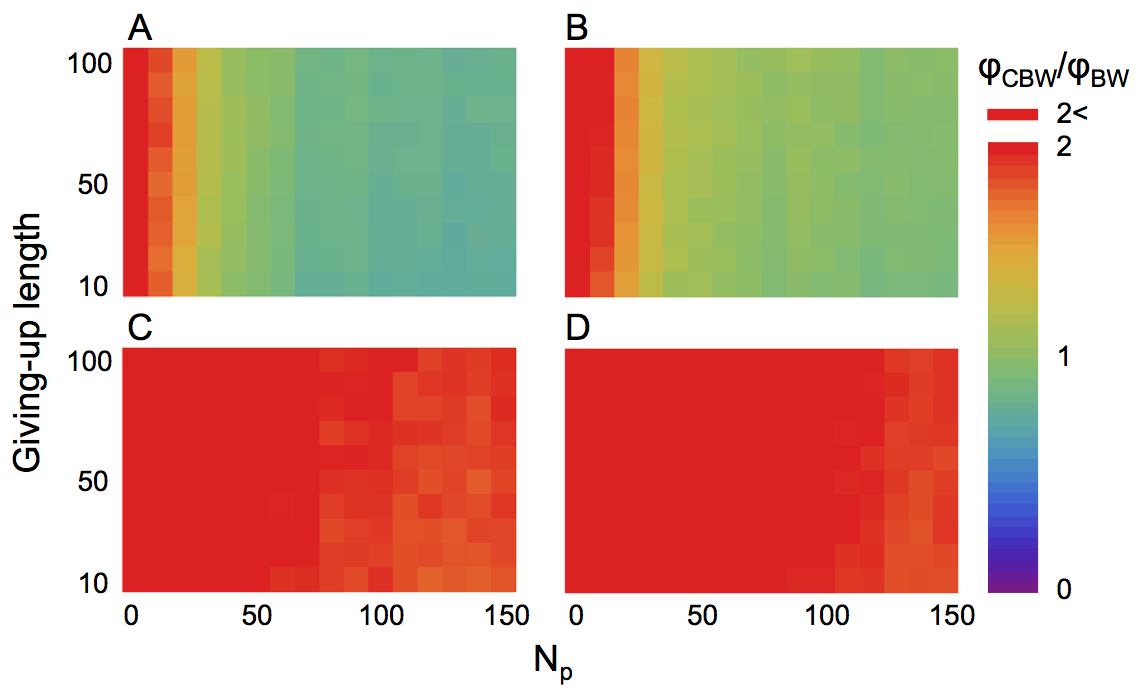

Supplement: S3 Fig — The strategy of predators is (A) sit-and-wait (v p = 0); (B) slow Lévy walker (v p/v s = 0.2); (C) middle Lévy walker (v p / v s = 1); and (D) fast Lévy walker (v p / v s = 5). The horizontal axis represents the number of predators introduced, and the vertical axis represents the giving-up length of the searcher. The total search time is 107 for sit-and-wait, slow, and middle predator conditions and 5×10^7^ for fast predator conditions. (TIFF) [file pcbi.1004601.s004.tiff]

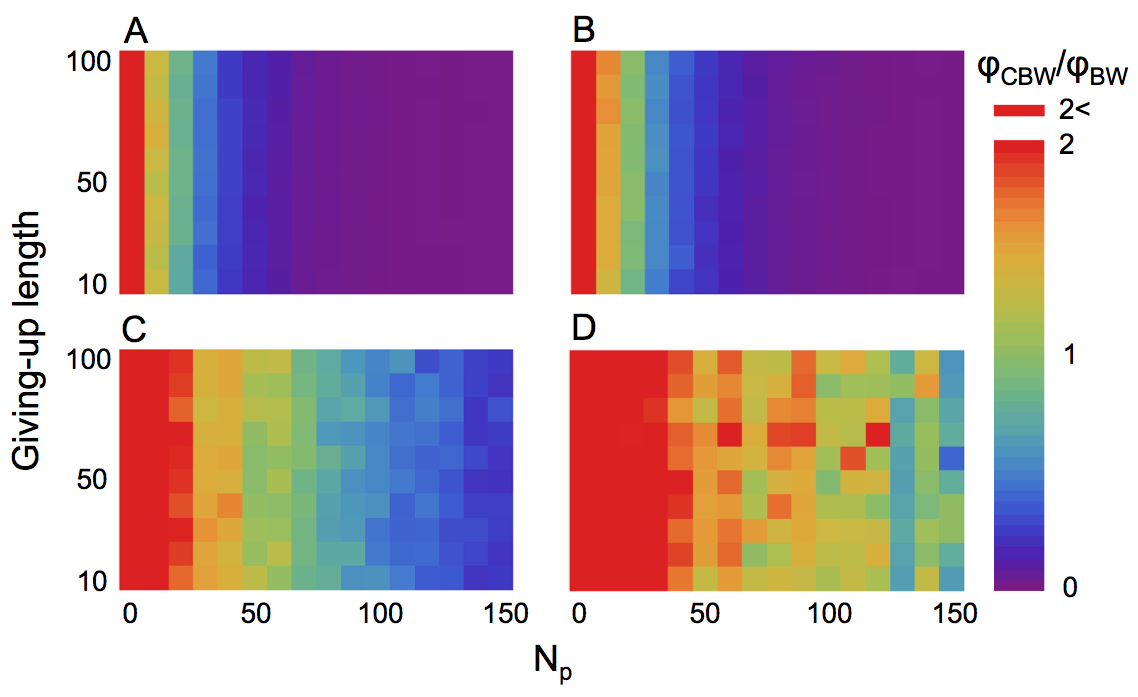

Supplement: S4 Fig — The strategy of predators is (A) sit-and-wait (v p = 0); (B) slow Lévy walker (v p / v s = 0.2); (C) middle Lévy walker (v p/v s = 1); and (D) fast Lévy walker (v p / v s = 5). The horizontal axis represents the number of predators introduced, and the vertical axis represents the giving-up length of the searcher. The total search time is 107 for sit-and-wait, slow, and middle predator conditions and 5×10^7^ for fast predator conditions. (TIFF) [file pcbi.1004601.s005.tiff]

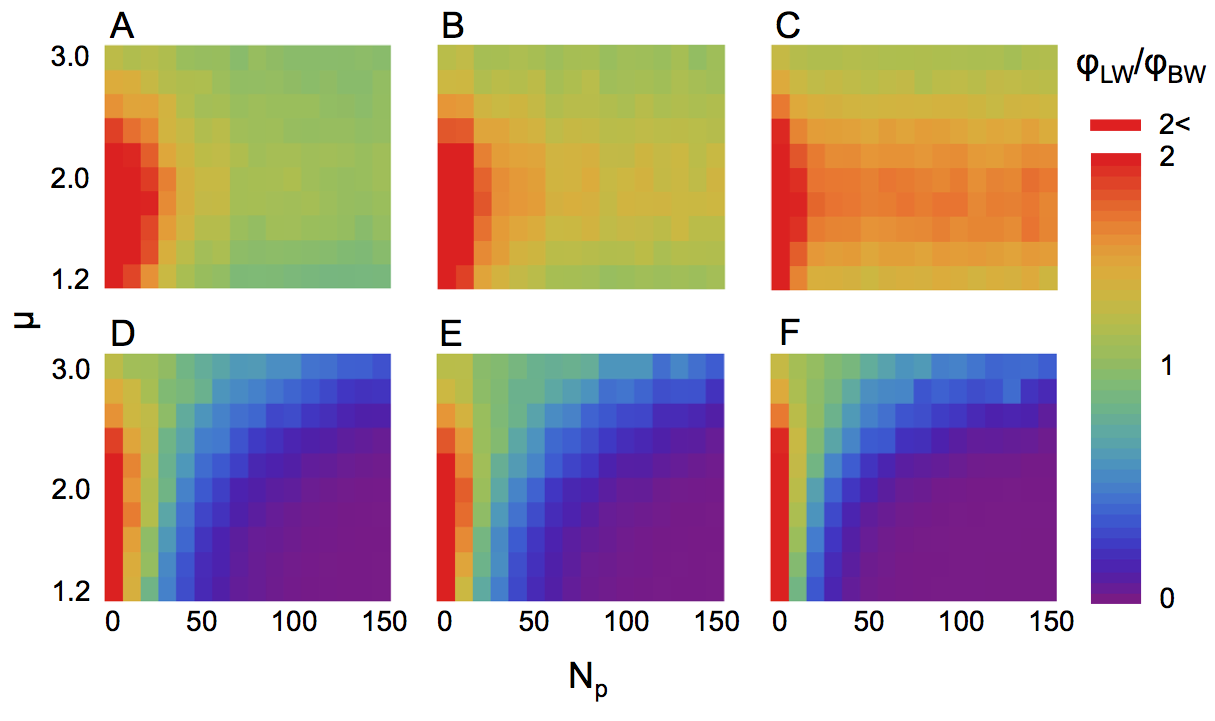

Supplement: S5 Fig — The strategy of predators is (A, D) slow Brownian walker (v p / v s = 0.2); (B, E) middle Brownian walker (v p/v s = 1); and (C, F) fast Brownian walker (v p / v s = 5). The horizontal axis represents the number of predators introduced, and the vertical axis represents the giving-up length of the searcher. The total searching time is 107 for sit-and-wait, slow, and middle predator conditions and 5×10^7^ for fast predator conditions. (TIFF) [file pcbi.1004601.s006.tiff]

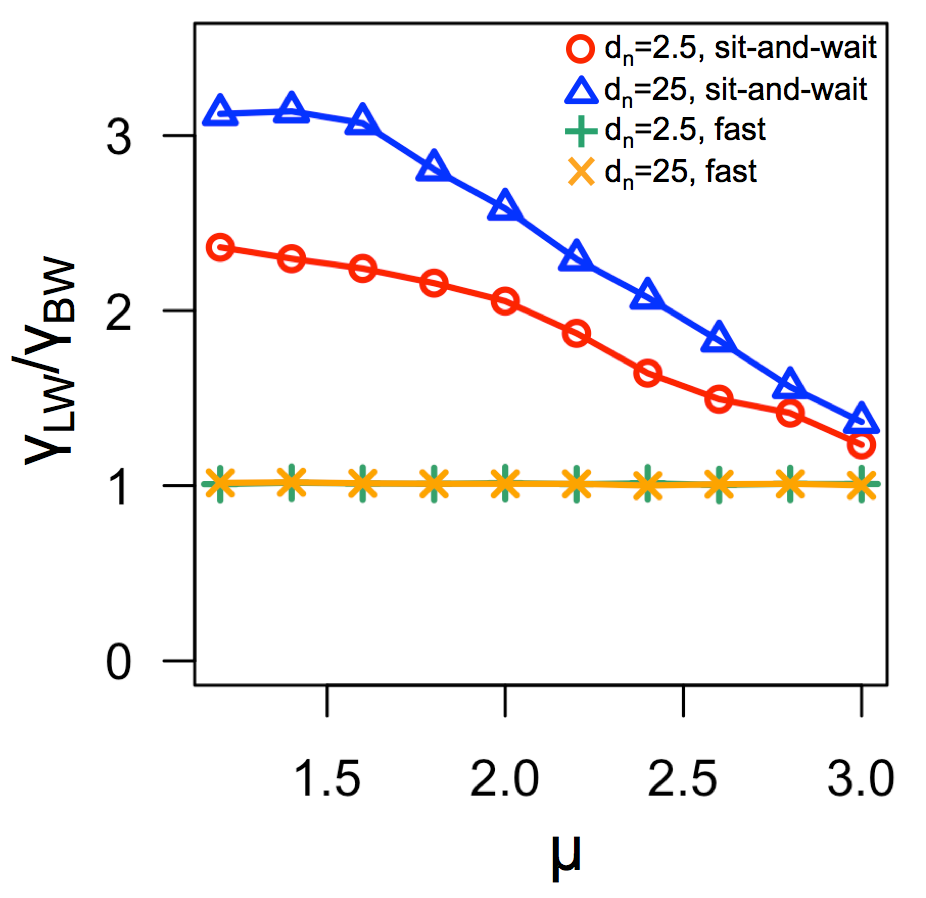

Supplement: S6 Fig — The horizontal and vertical axis represents the Lévy index μ of the searcher and the relative encounter rate γLWγBW with predators, respectively. When predators adopt a sit-and-wait strategy, the close distance to the nearest predator can lead to make γLWγBW low. The number of predators is 100 and other parameters are the same as those of the main results. (TIFF) [file pcbi.1004601.s007.tiff]
